# Energetic ions and photons for engineering nanomaterials

**DOI:** 10.3762/bjnano.17.82

**Published:** 2026-08-28

**Authors:** Devesh Kumar Avasthi, Venugopal Rao Soma

**Affiliations:** 1 Centre for Interdisciplinary Research and Innovation, UPES, Dehradun 248007, India; 2 DRDO Industry Academia-Centre of Excellence (DIA-CoE; formerly ACRHEM), University of Hyderabad, Hyderabad 500046, Telangana, India; 3 School of Physics, University of Hyderabad, Hyderabad 500046, Telangana, India

**Keywords:** energetic ions, ion beam/laser beam modification, ion beam–nanomaterial interactions, ion track, nanomaterials, surface nanopatterning, swift heavy ions

Over the last few decades several researchers have shown interest in nanomaterials, nanocomposites, two-dimensional (2D) materials, and nanostructured thin films due to their size-dependent chemical, physical, electrical, and optical characteristics, which are completely distinct from those of their bulk counterparts. Novel functional materials are key for various advancements in technologies wherein nanomaterials have a special role since the properties of the same material can be altered by varying the grain size in the nanometer regime typically from 1–100 nm. Ion beam/laser beam modification of different types of materials has been of great interest for engineering materials with desired/tailored properties as well as to understand the mechanism behind modification of materials [1]. Interestingly, energetic ions have the potential to synthesize and engineer/modify nanostructures. The interaction of energetic ions with nanoscale materials is different from that of bulk materials. Therefore, in the last two decades, it became a feature for fundamental understanding and ensuing applications to the scientific community [2–3]. Ion beams from accelerators have a wide spectrum of applications; starting from doping of semiconductors, since the early 1960s, which brought a revolution in semiconductor industries [4]. Other applications include ion beam therapy against cancer [5], an effective way of treating tumors with radiation causing minimum damage to near region tissues, radioactive isotopes for medical diagnostics [6], testing of integrated circuits of satellites against space radiation [7], simulating damage to future structural materials for fusion and fission reactors [8–10]. The energetic ions while going through materials interact via elastic and inelastic collisions, thereby losing their energy and are finally stopped at certain depth. In the low-energy regime, typically up to a few hundred keV, the energy is predominantly lost by elastic collisions and is referred as nuclear energy loss. The elastic collisions cause displacement of atoms resulting in vacancies. When the displaced atoms have an energy high enough to displace other atoms, it causes a collision cascade producing a cluster of defects. In the high-energy regime, typically greater than or equal to 1 MeV per nucleon of heavy ions, the impinging ions have velocity close to or higher than the Bohr electron velocity, the collisions of ions with the atoms of the material are inelastic. Thereby, the atoms in the material remain intact regarding their position, and are “silent spectators” of the excitation or ionization of atoms along the ion path. However, for energies beyond a threshold value, especially for insulators, a cylindrical defect is produced along the ion path, which is known as ion track [11]. Such high-energy heavy ions are known as swift heavy ions (SHI). The creation of an ion track is explained by the Coulomb explosion [12] and thermal spike [12–14] models. Low-energy ions are promising candidates for the synthesis of buried nanostructures of implanted species [15], compound phase nanostructures by ion-beam mixing [16], nanoripples [17], and nanodots [18] at the surface as well as for modifying the nanostructures. The nanoripples can act as templates for nanowires at the surface or aligned nanodots in arrays parallel to each other [19]. Focused ion beams, typically of tens of keV, are capable of creating any type of nanostructures, such as pillars, cones, cylinders, pyramids, and springs [20]. Ion tracks generated by SHI in polymers can be chemically etched to form narrow, hollow cylinders of diameters typically ranging from 10–100 nm or above [21]. These etched tracks have various applications [22]. The SHI irradiation results in change of nanoparticle diameter or elongation of nanoparticles embedded in a silica matrix in the ion direction, depending on the size of the particle with respect to the interparticle separation and the ion track diameter in the silica matrix [23]. The change in size of nanoparticles occurs when their size is smaller than the ion track diameter in the metal–silica nanocomposite film. The decrease in diameter occurs when the interparticle separation is large, whereas the increase in nanoparticle diameter occurs if the interparticle separation is small. The elongation of the nanoparticle occurs along the ion direction, when the size of the nanoparticle is equal or bigger than the diameter of the ion track [23]. Conducting carbon nanorods have been reported by SHI irradiation of diamond like carbon (DLC), fullerene films, and organic thin films [24–25]. Conducting nanorods are shown to have field emission properties [25]. SHI and low energy ions are being utilized for radiation tolerance studies of structural materials for fission as well as fusion reactors, where nanostructures have special relevance [9–10]. Most of the properties of the materials can be significantly altered by adjusting the input parameters of the ion and photon beams [26–28]. For instance, the sizes and shapes of particles contained in a silica matrix can be engineered using ion beams. The size and shape of the nanostructures produced can also be controlled by ultrashort laser pulses, which makes these materials suitable for a range of uses in various fields such as energy, defense/security, medical, and catalysis. A schematic depicting the various phenomena and diverse applications of energetic ions in materials science is presented in Figure 1.

**Figure 1:**
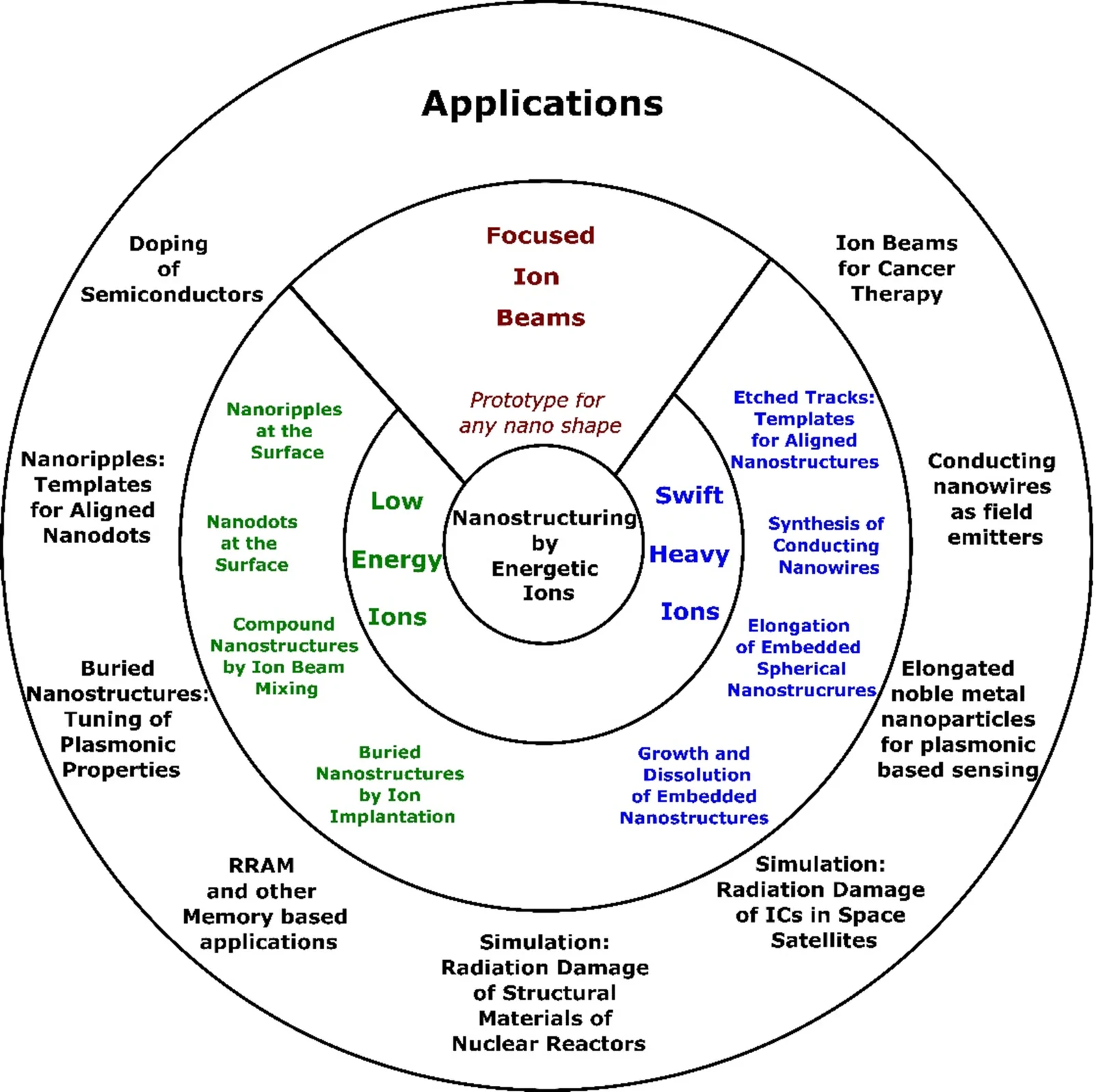
A schematic explaining the effects of ion beams in different energy regimes on various materials and the resulting applications.

Energetic ions and high-power laser pulses, due to their unique capability of depositing high energy density in ultrashort time scales (nanoseconds to femtoseconds), drive the materials to nonequilibrium states, similar to extreme conditions in localized zones at the nanoscale. This results in phase transformations, creation of defects, and modification of materials in terms of their optical, electrical, and mechanical properties. Therefore, the ion/laser irradiation is a unique tool to provide extreme conditions in a localized zone to test the designed materials for harsh environments. The studies on these aspects are critical for understanding and developing novel materials for extreme environments, such as nuclear reactors and space electronic devices. In addition, the energetic ions have other applications such as doping of semiconductors and ion-beam therapy against cancer.

The global laser market is a prominent industry, with sales reaching approximately USD $20–25 billion as of 2025, driven by diverse applications across manufacturing, medicine, and information technology [29–30]. In manufacturing, lasers are essential for processes such as cutting, welding, and three-dimensional additive manufacturing, utilizing high spatial coherence properties for precise material processing without any mechanical wear. The medical field employs lasers for a wide range of procedures, from vision correction by laser-assisted in situ keratomileusis (LASIK) and dermatology to specialized surgeries that minimize bleeding by sealing blood vessels during the cutting process. Furthermore, lasers underpin modern communications through high-speed fiber-optic data transmission and facilitate high-density (e.g., terabytes and petabytes in small volumes) optical data storage which could last for a long period of time without losing the original data fidelity. Beyond industry and health, lasers are pivotal in scientific research and advanced metrology, enabling extremely precise time measurements via optical clocks and detailed material characterization through techniques such as Raman spectroscopy and laser-induced breakdown spectroscopy [31–32]. They play a critical role in sensing technologies such as laser imaging and detection (LIDAR) for environmental monitoring and space exploration, as well as in cutting-edge fields such as quantum photonics, laser cooling, and microscopy/imaging. Military applications range from target designators to potential directed energy weapons, while future energy technology explores laser-driven nuclear fusion for electricity generation. Its application extends into space for inter-satellite communication and planetary missions, highlighting the role of lasers as a versatile tool in modern science and defense [31–32].

High power laser pulses are of special interest in emerging fields of novel functional nanomaterials and surface nanostructures (NSs) fabrication. Focused ultrashort laser pulses on the surface of any material (including semiconductors, metals, and dielectrics) in air, vacuum, and in liquids result in a variety of nanoparticles and surface nanostructures that can be used as nanomaterials for sensing and photonic applications [33–36]. The material within the focused beam is driven to extreme temperatures and pressures resulting in novel phases of matter in many cases. Theerthagiri et al. [27] provided with a summary of the basic knowledge and significance of the pulsed laser processes, including many functions and mechanisms involved in the creation of several types of nanomaterials, including metal nanoparticles, oxides, nonoxides, and carbon-based materials. The design and production of innovative pulsed laser-induced nanomaterials with fascinating characteristics for cutting-edge catalytic applications can be practically directed by this review. Sreekala et al. [28] discussed various laser-assisted nanocolloidal synthesis methods and related thin-film fabrication procedures, especially those used for the fabrication and characterization of devices in fields of photonics, energy, and sensing. Tumkur et al. [31] provided a succinct yet comprehensive overview of the parallel developments in the fields of lasers and materials processing, highlighting several important advancements over time along with a critical evaluation of the current state of the art. They also provided a roadmap of anticipated developments in these fields for the next 50 years. Yang et al. [33] presented a comprehensive review on the deeper physics understanding of ultrafast laser–matter interactions. This includes various description of nonlinear optical phenomena such as (i) multiphoton ionization, (ii) avalanche ionization, and (iii) laser-induced plasma. Furthermore, they reviewed how such interactions can help in achieving precise refractive index changes, meticulous ablation, and material structuring at the nanoscale in diverse materials (e.g., dielectrics, semiconductors, and metals). These phenomena govern the carrier excitation and subsequent energy deposition, resulting in structural modification. Venugopal Rao et al. [34] and Byram et al. [35] summarized recent developments in ultrafast laser ablation techniques for producing diverse metallic and semiconductor nanomaterials in the presence of various liquids. The advantage of ultrafast ablation in the presence of liquids is that it can produce nanoparticles/nanocolloids (in liquid) and surface microstructures/nanostructures on the target used for ablation.

This thematic issue discusses, in nine articles, several aspects of materials modification by energetic ions in a wide energy range (from a few tens of keV to hundreds of MeV) and high-power laser pulses. Moram et al. [37] investigated the effect of picosecond laser pulses and their wavelength along with the surrounding liquid on the formation of Ag, Au, Ag/Au nanoparticles (NPs) achieved using laser ablation in liquid. They prepared paper-based, flexible surface-enhanced Raman scattering (SERS) substrates using these NPs and demonstrated the detection of chemical warfare agent simulants such as methyl salicylate and dimethyl methyl phosphonate. Because of the fragmentation effects, their results demonstrated that the UV irradiation was far more advantageous for producing smaller nanoparticles. Das et al. [38] fabricated high-dielectric hafnium-based NPs and nanostructures using picosecond laser ablation of a hafnium metal target in three different liquid media (deionized water, toluene, and anisole) achieving HfO_2_ and HfC NPs along with Hf NSs. Interestingly, they observed core–shell type HfC nanoparticles when the ablation was performed in toluene and anisole. They also have successfully demonstrated the formation of laser-induced periodic surface structures (LIPSS) with low spatial frequency and high spatial frequency in the same experiment.

Mukherjee et al. [39] have studied the minutiae of an ultralow-energy magnetron-based electron cyclotron resonance ion source. In order to create ion beams in a microwave-coupled plasma-based ultralow-energy electron cyclotron resonance ion source, which is typically employed for nanostructuring solid surfaces, this article presented a thorough optimization of important parameters. Their study methodically investigated how important factors such as the gas pressure, magnetron power, extraction voltage, and ion energies affect the ion beam current. In addition to improving the knowledge and know-how of electron cyclotron resonance (ECR)-based ion sources, this work possibly creates opportunities for novel uses in the areas of materials science and nanotechnology. Bura et al. [40] reported 30 keV Ar+ implantation-induced fluence-dependent tailoring of radio frequency-sputtered ZnO films for the structural and optical properties. The crystallinity of the implanted ZnO films decreases with ion fluence. Implantation-induced structural modifications are correlated with the changes in diffuse reflectance, Urbach energy, and optical bandgap. The low reflectance values of implanted films have potential applications as transparent windows and anti-reflective coatings. Dutt et al. [41] present characterization of ion track-etched conical nanopores in SiO_2_ films (synthesized by thermal deposition and plasma-enhanced chemical vapor deposition (PECVD) methods) using synchrotron-based small angle X-ray scattering experiments. The ion tracks were created by high energy (89 MeV to 1.6 GeV) Au ions irradiation of amorphous SiO_2_ thin films. The conical nanopores were fabricated by chemical etching of the ion tracks in the SiO_2_ films. The nanopores have promising potential for applications such as filtration, sensing, and nanofluidics. This study revealed that nanopores in thermal SiO_2_ exhibits an exceptionally narrow size distribution of only 2–4%, as compared to that in PECVD deposited films. It was observed that there are substantial differences between the nanopores in thermal and PECVD SiO_2_ films. Loeber et al. [42] reported on using focused ion beam-induced platinum deposition with a low-temperature cesium ion source and compared it with that of conventional Ga ion source. Different acceleration voltages and ion beam currents were required by the two ion sources. The deposition rate is found to linearly depend on the current density. The Cs ion source is an emerging new source having higher milling rate due to its heavier mass than that of Ga. Chaudhary et al. [43] studied the formation of laser-induced periodic surface structures (LIPSS) on a stainless-steel surface using femtosecond laser pulses with wavelengths spanning from 400 to 2400 nm. They observed that the penetration depth increased with increasing wavelength (up to 2000 nm). The value reached a peak at ≈13 µm and, subsequently, it decreased. Their research results demonstrated that the periodicity attained at each wavelength and the laser penetration depth were correlated. They claim that their studies offer important insights into the mechanics and optimization of the LIPSS production on stainless steel surfaces. Jany et al. [44] investigated the ion-beam-induced decomposition of four different Cu/Ag metal-organic precursors when they were exposed to 30 keV gallium focused ion-beam (FIB) irradiation. This investigation is of importance as modern metal deposition techniques, such as focused ion-beam-induced deposition (FIBID) heavily relies on the availability of metal-organic precursors of particular properties. They analyzed the chemical composition along with the morphology of the resulting structures. They claim that their approach yields insightful information on the physics of underlying metal deposition from different metal-organic precursors. Bist et al. [45] studied the WC–WO_3_ nanocomposite coating synthesized by sputtering at room temperature followed by annealing at 700 °C for its potential application as coating of a plasma-facing wall of a fusion reactor. It is shown by ion irradiation and sputtering measurements that the coating has promising properties in terms of lower sputtering rate as well as low radiation damage. The heterogeneous interfaces act as vacancy sinks leading to lower radiation damage whereas the high binding energy of W in WO_3_ is responsible for the observed lower sputtering rate.

Devesh Kumar Avasthi and Venugopal Rao Soma

Dehradun and Telangana, August 2026

## Data Availability

Data sharing is not applicable as no new data was generated or analyzed in this study.
